# Alternative blood transfusion triggers: a narrative review

**DOI:** 10.1186/s12871-024-02447-3

**Published:** 2024-02-23

**Authors:** Ardak Arynov, Dilyara Kaidarova, Barbara Kabon

**Affiliations:** 1 Department of Anesthesiology and Intensive Care, Kazakh Institute of Oncology and Radiology, Abay av. 91, Almaty, Kazakhstan; 2 Kazakh Institute of Oncology and Radiology, Abay av. 91, Almaty, Kazakhstan; 3 Department of Anaesthesia, General Intensive Medicine and Pain Medicine Medical, University of Vienna, Spitalgasse 23, 1090 Vienna, Austria

**Keywords:** Anemia, Blood transfusion, Transfusion triggers, Oxygen delivery, Oxygen consumption

## Abstract

**Background:**

Anemia, characterized by low hemoglobin levels, is a global public health concern. Anemia is an independent factor worsening outcomes in various patient groups. Blood transfusion has been the traditional treatment for anemia; its triggers, primarily based on hemoglobin levels; however, hemoglobin level is not always an ideal trigger for blood transfusion. Additionally, blood transfusion worsens clinical outcomes in certain patient groups. This narrative review explores alternative triggers for red blood cell transfusion and their physiological basis.

**Main Text:**

The review delves into the physiology of oxygen transport and highlights the limitations of using hemoglobin levels alone as transfusion trigger. The main aim of blood transfusion is to optimize oxygen delivery, necessitating an individualized approach based on clinical signs of anemia and the balance between oxygen delivery and consumption, reflected by the oxygen extraction rate. The narrative review covers different alternative triggers. It presents insights into their diagnostic value and clinical applications, emphasizing the need for personalized transfusion strategies.

**Conclusion:**

Anemia and blood transfusion are significant factors affecting patient outcomes. While restrictive transfusion strategies are widely recommended, they may not account for the nuances of specific patient populations. The search for alternative transfusion triggers is essential to tailor transfusion therapy effectively, especially in patients with comorbidities or unique clinical profiles. Investigating alternative triggers not only enhances patient care by identifying more precise indicators but also minimizes transfusion-related risks, optimizes blood product utilization, and ensures availability when needed. Personalized transfusion strategies based on alternative triggers hold the potential to improve outcomes in various clinical scenarios, addressing anemia’s complex challenges in healthcare. Further research and evidence are needed to refine these alternative triggers and guide their implementation in clinical practice.

## Introduction

Anemia is a clinical hematological syndrome which is characterized by low levels of hemoglobin and, in most cases, red blood cells (RBCs) [1]. The World Health Organization (WHO) identifies anemia as a global problem for public health and according to an estimation using prevalence data from 1993–2005, 24.8% of the global population was affected by this pathology. In particular, anemia is concentrated in children, pregnant and non-pregnant women [2]. The global anemia prevalence in 2010 was 32.9% (more than 2.2 billion people) and the most common cause of anemia was iron-deficiency [3].Thus, anemia is considered to be the most common hematological syndrome in clinical practice.

According to the WHO classification, anemia is defined as a hemoglobin concentration below 129 g/l in men and 119 g/l in women at sea level (Table 1) [4].

**Table 1:** Hemoglobin levels to diagnose anemia at sea level (g/l)

Population	Non-Anemia *	Anemia		
		mild	moderate	severe
Children aged 6–59 months	110 or higher	100–109	70–99	Lower than 70
Children aged 5–11 years	115 or higher	110–114	80–109	Lower than 80
Children aged 12–14 years	120 or higher	110–119	80–109	Lower than 80
Non-pregnant women (aged 15 years and above)	120 or higher	110–119	80–109	Lower than 80
Pregnant women	110 or higher	100–109	70–99	Lower than 70
Men (aged 15 years and above)	130 or higher	100–129	80–109	Lower than 80

Anemia in the perioperative period and in critically ill patients is a particular problem. The rate of preoperative anemia depends on age, sex, comorbidity and surgical pathology and can reach up to 75% [5, 6]. Despite such a high prevalence in clinical practice, preoperative anemia has long been perceived as a relatively minor problem that can be easily eliminated by blood transfusion [7]. Meanwhile, numerous clinical trials suggest that preoperative anemia is associated with poor outcomes and increased mortality in both cardiac and non-cardiac surgery patients [8–12]. Transfusion of RBCs remains the most common approach for perioperative anemia treatment. Thus, the American Red Cross reports that nearly 16 million blood components units are transfused annually in the USA, with approximately 29,000 units of RBCs needed daily [13].

**Table 2:** Clinical trials demonstrating the impact of blood transfusion on clinical outcomes

Trials	Study population	Conclusion
Smilowitz N.R. et al. [14] Retrospective cohort study	3,050 patients underwent orthopedic surgery	Blood transfusion was associated with increased mortality.
Glance L.G. et al. [15] Retrospective study	10,100 patients undergoing general, vascular or orthopedic surgery	Intraoperative RBC transfusion was associated with increased risk of mortality and morbidity (septic, thromboembolic complications, etc.)
Bernard A.C. et al. [16] Prospective systematic study	125,223 general surgery patients in 121 hospitals as part of the ACS-NSQIP (The American College of Surgeons National Surgical Quality Improvement Program)	Intraoperative blood transfusion (even one unit of packed RBC) was associated with increased risk of mortality and morbidity in general surgery patients.
Woldendorp K, et al. [17] Meta-analysis of thirty-nine studies	Thirty-nine studies with 180,074 patients after cardiac surgery	Perioperative red blood transfusion appeared to be associated with a significant reduction in long-term survival for patients after cardiac surgery.
Li Y et al. [18] Retrospective study	6,752 patients who underwent cardiac surgery	Blood transfusion was associated with worse outcomes in cardiac surgery patients
Dae won Park et al. [19] Propensity-matched analysis of a prospective observational database	1,054 patients severe sepsis and septic shock.	Red blood cell transfusions were associated with lower risk of mortality
Suk Yong Jang et al. [20] Nationwide Cohort Study	10,973 patients in the transfusion group and 3,771 patients in the non transfusion group	Transfusion group did not show significantly worse results than the non-transfusion group
Wen-Chih Wuet al [21] retrospective study	Patients with acute myocardial infarction, 65 years and older	Blood transfusion is associated with a lower short-term mortality rate
Yi Zheng et al. [22] a systematic review and meta-analysis	28,797 patients in intensive care unit	RBC transfusion does not increase the risk of in-hospital mortality

As shown in Table 2, blood transfusion has varying effects on clinical outcomes—both improvement outcomes and an increase in the risks of adverse outcomes. A similar situation arises in studies comparing restrictive and liberal transfusion strategies. For example, in the randomized ‘Transfusion Requirements in Critical Care (TRICC)’trial, a restrictive strategy showed a similar 30-day mortality compared to a liberal strategy in critically ill patients. However, mortality in the restrictive group was significantly lower among the subgroups- patients with Acute Physiology and Chronic Health Evaluation II score of ≤  20 and younger patients (less than 55 years) and was also lower during hospitalization. Nevertheless, there was no difference in mortality among patients with cardiac pathology [23]. In accordance, another multicenter randomized trial demonstrated an identical mortality rate in septic shock patients between a liberal and restrictive transfusion strategy. The number of patients experiencing ischemic events (including cerebral ischemia and acute myocardial ischemia), adverse transfusion reactions and requiring life support were comparable in both groups [24]. Additionally, more recently, a systematic Cochrane database review analysed 48 trials and showed that a restrictive blood transfusion strategy led to a 41% reduction in the proportion of individuals subjected to red blood cell transfusions [25]. Authors suggested that there is no evidence of influence of the restrictive strategy on mortality and morbidity (such as stroke and myocardial infarction). Nevertheless, the authors emphasized that in some patient groups, higher hemoglobin levels may be preferable, but there was insufficient evidence to recommend applying a specific strategy to certain patient subgroups (traumatic brain injury, myocardial infarction, etc). Importantly, the authors were of the conviction that hemoglobin levels do not always reliably reflect the need for transfusion in some patients. A further large meta-analysis also demonstrated a reduction in in-hospital mortality among critically ill patients treated with a restrictive transfusion strategy [26]. On the other hand, a liberal blood transfusion strategy improved survival and decreased complications specifically in elderly patients and patients with cardiovascular diseases [27–29]. This issue is well described by Patel and co-authors, who, in a systematic review and meta-analysis, compared both cardiac and non-cardiac randomized clinical trials, as well as observational studies, investigating restrictive and liberal strategies [30]. The authors demonstrated that the results of randomized studies in cardiac surgery were not in accordance with the a general assumption that a liberal strategy significantly increases mortality and morbidity. Thus available evidence supports the inconsistency of the hemoglobin level as a transfusion trigger, especially in specific cohorts of patients. However, several modern guidelines suggest using the hemoglobin level as the only blood transfusion trigger and in most cases recommend a restrictive strategy [31–33].

The main aim of blood transfusion administration is an optimization of oxygen delivery rather than to approach a certain level of hemoglobin. Consequently, many authors and guidelines suggest an individualized approach to blood transfusions, based not only on the level of hemoglobin (which often cannot clearly reflect the need for oxygen delivery), but also on the clinical signs of anemia and the balance between oxygen delivery and oxygen consumption [32, 34–36].

One of the indications for blood transfusion may also be one of its physiological effects - increasing intravascular volume and, consequently, increasing blood pressure [37]. Thus, sustained perioperative hypotension and increased vasopressor support might also represent a trigger for a more liberal administration of blood components. The concept of physiological triggers for blood transfusion is based on the principles of anemia correction in terms of the oxygen delivery/consumption ratio.

The aim of our narrative review is to summarize the current role of alternative triggers for RBCs transfusion, such as the oxygen extraction ratio, blood lactate level, central venous/mixed venous oxygen saturation, oxygen content arterial-venous difference and ischemic signs on the electrocardiogram. Thus, we provide insights into the physiology of oxygen transport and emphasize the importance of oxygen delivery optimization regarding decision making in transfusion therapy. We give an overview of studies that which explore alternative triggers and their limitations in clinical practice and current blood transfusion guidelines.

## Main text

### Physiological basis of oxygen transport

**Fig. 1:**
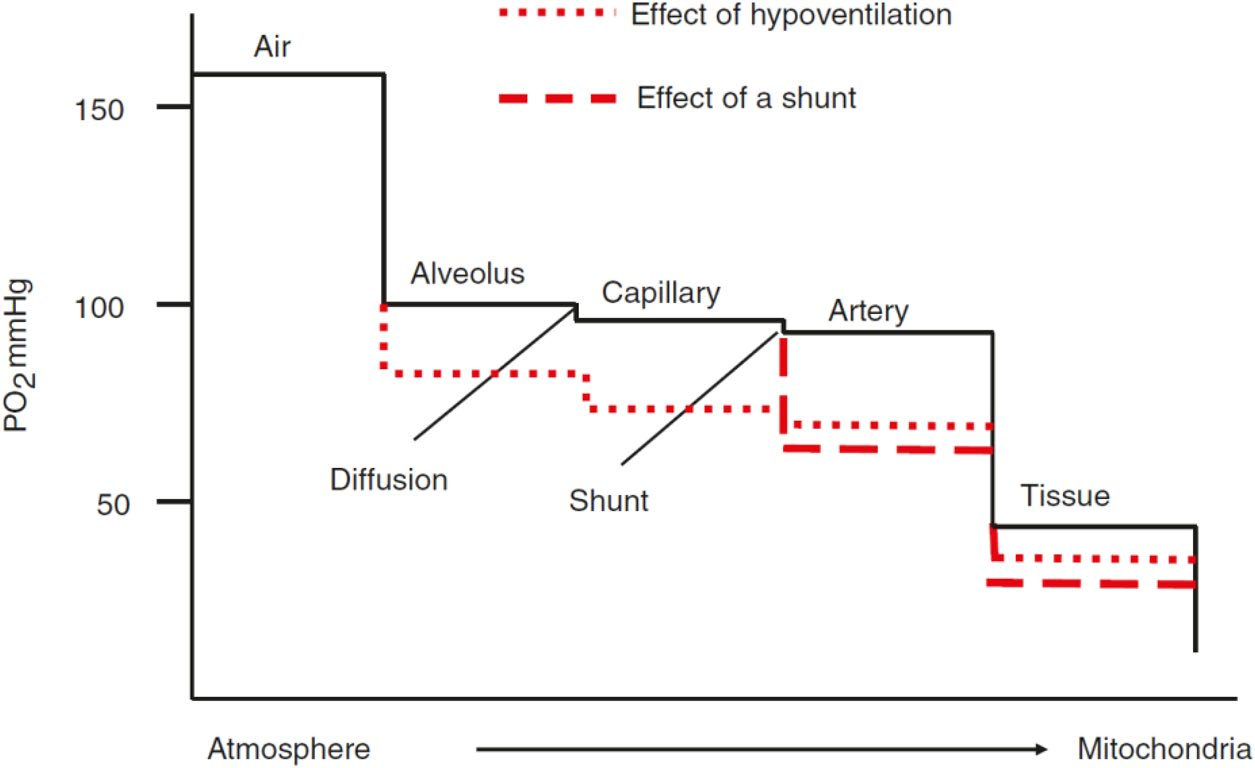
Oxygen cascade (Explanation in text. With the permission from: Springer Nature, License Number 5,666,460,857,294) [39]

Oxygen, discovered by Joseph Priestley in 1772, is the eighth element according to the serial number in the periodic system [38]. Also among the pioneers and researchers of oxygen were renowned scientists Carl Wilhelm Scheele and Antoine-Laurent Lavoisier. Oxygen plays an integral role in cellular respiration and is, therefore, essential for the life of all aerobic organisms. Oxygen partial pressure (tension) (PO_2_) in the atmospheric air at sea level fluctuates within 150–160 mmHg, while the transport chain of oxygen to the target cells is characterized by a physiological stepwise decrease in oxygen tension from inhaled air to the mitochondria. This phenomenon is called the “oxygen cascade” (Fig. 1) [39]. At the onset of inspiration the progress of declining oxygen tension occurs due to tracheal air humidification and alveolar carbon dioxide admixture and subsequently due to diffusion of oxygen through the alveolocapillary membrane and venous admixture and shunting [40, 41]. The partial pressure of oxygen continues to decrease in accordance with significant diffusion gradients between the capillaries and the cytoplasm of cells and finally the mitochondria. Current measurements demonstrate that tissue PO_2_ of most organs is in the range of 20–40 mmHg under resting conditions [42]. At rest and under physiologic conditions, oxygen delivery to the tissue still exceeds oxygen consumption by far with an oxygen extraction ratio near 25% [43]. However, the physiologic decrease of cellular oxygen levels can be further aggravated by pathophysiological states, as hypoventilation or ventilation/perfusion mismatch, that will finally result in tissue hypoxia.

Most of the oxygen (98–99%) binds to hemoglobin, while only 1–2% of the total amount of oxygen is dissolved in the plasma and does not play a significant role in the global oxygen delivery.

The term “total oxygen content” (**СtO**_**2**_) indicates the sum of the volumetric concentrations of both bound and dissolved oxygen in plasma [43]:

1 $${\textbf{CtO}}_{{\textbf{2}}}({\textbf{ml O}}_{{\textbf{2}}}/{\textbf{dl}})=({\textbf{1,34}} \times {\textbf{Hb}}\times {\textbf{SO}}_{{\textbf{2}}})+ ({\textbf{0,031}}\times {\textbf{PO}}_{{\textbf{2}}}),$$

*1,34- Huffner’s constant - describes the volume of O*_*2*_ *(ml) that binds 1 gram of hemoglobin at full saturation, 0,031- the Bunsen coefficient reflects the volume of oxygen that dissolves in 1 L of plasma for every 1 mmHg of partial pressure, Hb- hemoglobin concentration, g/l, PO*_*2*_ *- partial pressure of oxygen, SO*_*2*_ *- hemoglobin oxygen saturation. To calculate this indicator in arterial or venous blood, it is necessary to use the corresponding SO*_*2*_ *and PO*_*2*_ *values.*

As formula (1) shows, PO_2_ equal to100 mmHg can only provide 3 ml of dissolved oxygen in 1 L of blood, while a saturation of 99% means that 134 ml of oxygen is bound at a hemoglobin level of 100 g/L. This clearly demonstrates the low possibility of increasing oxygen delivery in patients with anemia.

Oxygen delivery is determined by the following formula [43]:

2 $${\textbf{{DO}}}_{\textbf{{2}}}\hspace{0.17em}=\hspace{0.17em}{\textbf{{CO}}}_{\textbf{2}}\times {\textbf{{CO}}},$$

DO_2_ - oxygen delivery, ml/min, CаO_2_ – total oxygen content in arterial blood, ml/l, CO- cardiac output, liters per minute.

Oxygen consumption is calculated using the following formula [43]:

3 $${\textbf{{VO}}_{2}}= ({\textbf{Cta}}{\textbf{O}}_{\textbf{2}} - {\textbf{Ctv}}{\textbf{O}}_{\textbf{2}}) \times {\textbf{CO}},$$

VO_2_– oxygen consumption, ml/min, CvO_2_ – total oxygen content in mixed venous blood, ml/dl.

Oxygen consumption is also expressed as an oxygen extraction ratio (O_2_ER) or an oxygen extraction index [43, 44]:

4 $${\textbf{O}}_{\textbf{2}}{\textbf{ER}}=\frac{{VO}_{2}}{{DO}_{2}}\times {\textbf{100}}{\textbf{\%}}=\frac{{CtaO}_{2}-{CtvO}_{2}}{{CtaO}_{2}}\times {\textbf{100}}\varvec{\textbf{\%}},$$

*O*_*2*_ *ER- oxygen extraction ratio, %, CаO*_*2*_ *– total oxygen content in arterial blood, ml/dl; CvO*_*2*_ *- total oxygen content in mixed venous blood, ml/dl.*

Normal values of these indicators are 20–30%.

### Concepts of tissue oxygen delivery

#### Anemia and oxygen delivery

According to the classification proposed by Joseph Barcroft in 1920, anemia leads to anemic hypoxia associated with decreased blood oxygen content. According to this classification other types are hypoxemic hypoxia due to lack of oxygen in the inhaled air or ventilation disorders and circulatory hypoxia - associated with acute and/or chronic heart failure [45]. A few years later this classification was supplemented with the fourth type named histotoxic (tissue) hypoxia by J.P. Peters and D.D. Van Slyke, which is based on the blockade of respiratory enzymes [46]. Hemoglobin in red blood cells has been traditionally viewed as a passive transport system for oxygen and the main determinant of oxygen deliverly. Nevertheless, adequate local blood flow and thus tissue oxygen delivery are largely defined by the ability of the cell to generate adenosine 5′-triphosphate ATP from oxygen. One important fact of oxygen transport physiology is the independence of oxygen consumption upon oxygen delivery:oxygen consumption is constant over a wide range of DO_2_ under resting conditions, and decreases only when DO_2_ falls below a critical level (DO_2_crit) [47–49].

In this context overall oxygen consumption remained stable in healthy resting humans during severe acute limitation of oxygen delivery due to isovolemic reduction of hemoglobin concentration to various low levels (Table 3).

However, globally maintained oxygen consumption does not reflect the heterogenous difference inorgan specific oxygen supply and demand, and various organ systems might have different tolerances for anemia [50].

#### Adaptive responses to anemia

The first line of compensation for reduced oxygen delivery is increased blood flow through the tissues i.e. increased cardiac output [51]. During anemia an increase in cardiac index and heart rate associated with a sharp increase in myocardial oxygen consumption maintains global overall systemic oxygen delivery [52]. Given a constant systemic oxygen use, this fact requires that other organs, such as the gut or the kidney, must actually reduce oxygen consumption [51, 53].Thus organs of a higher metabolic demand namely the heart and the brain receive a greater proportion of blood flow and oxygen delivery [54]. Due to a limited blood flow response to anemia of the renal tissue, renal hypoxia is proportional to the degree of anemia and at comparable hemoglobin levels, more severe levels of tissue hypoxia are observed in the kidney, relative to the brain. In this context the kidney serves as an early central oxygen sensor activating local responses including the increased production of serum erythropoietin as well as adaptive cardiovascular responses in order to protect vital organ perfusion [55, 56].

In contrast, the organism cannot compensate for a reduced oxygen delivery due to a decreased cardiac output, as there are no physiological mechanisms available to increase the hemoglobin level or the oxygen saturation above a certain given level. Even an increase of red blood cells might than not be sufficient to increase oxyden delivery adequately [57]. It is also important to remember that elevating cardiac output may be unsafe for patients with decreased cardiac functional reserve as commonly observed in (patients with cardiovascular diseases or of advanced age). Moreover the majority to these specific patients receive ß-blocker treatment, which has been shown to attenuate the cardiovascular response to acute anemia and to accentuate the risk of brain and heart ischemia [58, 59].

In anemia, arterial oxygen tension remains almost unchanged. However, anemia leads to a decrease in blood oxygen content, which is compensated by an increase in cardiac output and an increase in oxygen extraction by tissues associated with a shift in the oxyhemoglobin dissociation curve to the right by increasing the concentration of 2,3-diphosphoglycerate [60, 61]. It is important to note that an increase in the level of 2,3- diphosphoglycerate and, accordingly, a decrease in the affinity of hemoglobin for oxygen is a reaction that is typical for all types of anemia including chronic anemia [61, 62]. In healthy volunteers a decreased tissue oxygen availability with a concomitant increased oxygen extraction and a reduced mixed venous oxygen saturation was present at hemoglobin levels below 6 g/dL. Nevertheless, this hemoglobin concentration did not result in inadequate oxygen transport asassessed by plasma lactate concentration [52].

#### Critical level of oxygen delivery

**Fig. 2:**
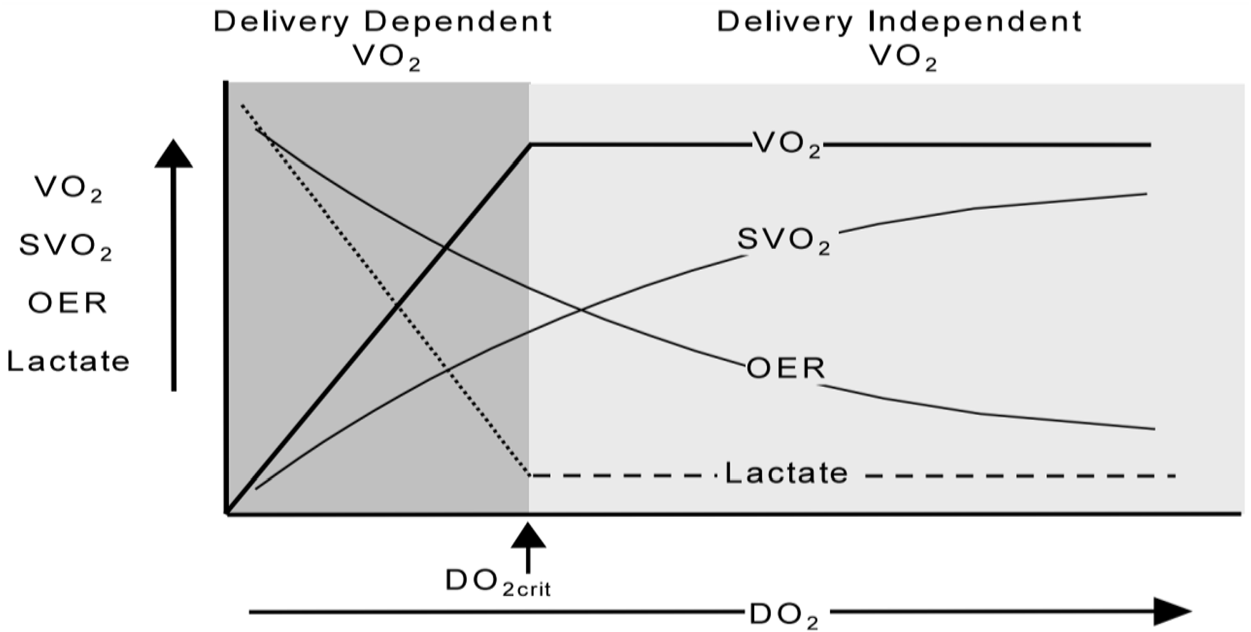
Relationship between delivery and consumption of oxygen. There is an increase in the level of lactate and a further increase in oxygen extraction with a decrease in the saturation of hemoglobin in venous blood when reaching the critical oxygen delivery (DO_2_- oxygen delivery, VO_2_- oxygen consumption, SvO_2_-saturation of hemoglobin in mixed/central venous blood, OER-oxygen extraction ratio, DO_2crit_- critical level of oxygen delivery)(With the permission from Elsevier Publisher, License Number 5,633,500,709,980) [64]

When the level of oxygen delivery decreases, oxygen consumption is provided by an increase in oxygen extraction [57, 63]. The development of oxygen debt is indicated by an increase in the level of blood lactate associated with a further increase in oxygen extraction and a decrease in the saturation of hemoglobin in the venous blood (Fig. 2) [64].

This level of critical oxygen delivery can vary under anemic conditions, as indicated by both clinical and experimental investigations. A clinical case that gave impetus to this concept was presented already some decades ago [65]. The report refers to an 84-year-old male patient with stomach cancer who underwent gastrectomy complicated by excessive bleeding with a blood loss of 4500 mL. The patient refused blood transfusions for religious reasons and died 12 h after surgery. The patient was invasively monitored with a pulmonary artery catheter and under conditions of hypervolemic hemodilution, a critical level of oxygen delivery-4.9 ml/kg per minute at a hemoglobin level of 40 g/l was identified. In this case oxygen consumption dependence upon delivery did not develop before at a very low level of hemoglobin has been reached. However, experimental studies showed various levels of hemoglobin that led to critical oxygen delivery in anemia (Table 3). The presented data demonstrate the imperfection of hemoglobin as a trigger for transfusion in specific clinical situations.

**Table 3:** Studies devoted to determining the critical level of DO_2_ in anemia

Trials	Population	Monitored indicators
J.A. Liebermanet et al. [66]	Healthy volunteers aged 19–25; acute normovolemic hemodilution, hemoglobin reduced from 12.5 ± 0.8 to 4.8 ± 0.2 g/dl	Identification of the critical level of oxygen delivery: DO_2_crit = 7,3 ml/min/kg
J.M. Leung et al. [67]	55 healthy volunteers; acute normovolemic hemodilution, hemoglobin level 5 g/dl	Assessment of myocardial oxygen delivery and consumption imbalance based on ST-segment changes: three volunteers had ST-segment changes on the background of tachycardia, without clinical symptoms and completely reversible.
D.R. Spahn et al. [68]	20 patients over 65 years without concomitant diseases of the cardiovascular system, acute normovolemic hemodilution, hemoglobin level 88 ± 3 g/l	Assessment of CI, O_2_ER, ST segment changes: the decrease in hemoglobin was compensated by the increase in CI and O2ER, there were no changes in the ST segment in lead II, and a slight decrease in the ST segment was noted in lead V5. There were no electrocardiographic signs of myocardial ischemia.
D.R.Spahn et al. [69]	60 patients with coronary artery disease who took long-term beta-blockers and underwent coronary artery bypass grafting	The decrease in hemoglobin to 99 ± 2 g/l was compensated by an increase in CI and O2ER; there were no electrocardiographic signs of myocardial ischemia.

* DO_2_crit – critical level of oxygen delivery- the level at which oxygen consumption begins to depend on its delivery, ml/min/kg; VO_2_– Oxygen Consumption, ml/min; Hb- hemoglobin level; CI- cardiac index, l/m^2^ per minute; O_2_ER – oxygen extraction ratio, %

As seen in Table 3, the hemoglobin level at which critical oxygen delivery is achieved varies and depends on age and comorbidities. It is important to understand that reaching the critical level of oxygen delivery in anemia is associated with a significantly increase in mortality. This is well demonstrated in a study of patients with anemia who did not undergo blood transfusion (patient refusals for religious and other reasons) [70]. Moreover, it has also been shown that reaching the critical level of oxygen delivery in anemia leads to a reduction in time to death and a limited time for intervention [70, 71].

Nevertheless, a strong association between decreased hemoglobin values and adverse events and mortality has also been established even before a critical level of oxygen delivery has been reached. Specifically, mortality rises sharply as hemoglobin decreases below 5-6 g/dl [72], while oxygen consumption may become dependent on delivery at even lower hemoglobin levels. Thus in clinical practice anemia treatment usually is initiated already well above this critical threshold and anemia induced organ injury due to a reduction of organ specific tissue oxygenation might be a more relevant mechanism for unfavourable outcomes.

### Blood transfusion triggers

As noted above, the main and only purpose of blood transfusion is to increase the oxygen– binding capacity of the blood and, as a result, oxygen delivery to organs and tissues. From a physiological point of view, the isolated hemoglobin level does not always reflect adequate oxygen delivery, which became the basis for the search for alternative blood transfusion triggers.

Modern requirements for an “ideal trigger” include not only high sensitivity and specificity and the possibility of its continuous monitoring, but also the ability to reflect the degree of anemia compensation by the body, contributing to timely and reasonable blood transfusions [73].

In the 1980s, an expert consensus, published in the Journal of the American Medical Association with relatively liberal approaches to blood transfusion, clearly states the need for an individualized approach in prescribing blood transfusion – assessment of clinical parameter, mixed venous blood oxygen tension, oxygen extraction level, and cardiac output [74].

Further studies in search of an alternative blood transfusion trigger suggested using the saturation of the level of oxygen extraction, central venous/mixed venous oxygen saturation, oxygen content arterial-venous difference, electrocardiogram, serum lactate and others.

### Oxygen extraction ratio

The oxygen extraction ratio reflects oxygen consumption by the body, while, as mentioned above, its extraction increases with a decrease in the oxygen delivery level, which ensures oxygen consumption at a constant rate. The level of oxygen extraction is one of the laboratory indicators of anemic hypoxia [75]. This indicator has demonstrated diagnostic value primarily in reducing the number of blood transfusions and served as a trigger for blood transfusions in a number of studies [75–79]. Furthermore, the significance of this indicator as a criterion for blood transfusion prescriptions was demonstrated in earlier and experimental studies [80, 81]. It should be noted that no large randomized trials of a blood transfusion strategy based on oxygen extraction level assessment have been conducted.

### Central venous/mixed venous oxygen saturation

Saturation of hemoglobin in mixed venous blood (SvO_2_) is one of the indicators reflecting the balance between oxygen delivery and consumption [82]. From a physiological point of view, SvO_2_ is considered an indicator that reflects the extraction of oxygen and disturbances in the ratio between its delivery and consumption [83]. The concept of using SvO_2_ as a blood transfusion trigger was mostly developed in the works of B. Vallet et al. Studies based on the physiological rationale demonstrated high sensitivity and specificity of SvO_2_ as a blood transfusion trigger [84–86].

However, the use of mixed venous blood hemoglobin saturation is associated with an invasive method (pulmonary artery catheter insertion) and high cost. In this regard, a possible alternative is the use of the hemoglobin saturation of the central venous blood (blood obtained from the mouths of the vena cava, ScvO_2_) [87]; normal SvO_2_ values are 68–77%, and ScvO_2_ is approximately 5% higher [88]. In a recent study, N. Themelin and coauthors assessed changes in systemic oxygen delivery and consumption after transfusions of donor RBCs and the diagnostic value of ScvO_2_ in patients in the ICU [89]. The authors have evaluated central venous blood hemoglobin saturation, blood lactate level, venous-to-arterial carbon dioxide (CO_2_) tension difference and cardiac index. Only hemoglobin saturation of the central venous blood with a baseline value of less than 70% showed positive changes after blood transfusion, while the effect on clinical outcomes remains unclear. It is necessary to remember that a decrease in SvO_2_(ScvO_2_) can develop with a decrease in arterial oxygenation (hypoxic hypoxia), a decrease in cardiac output (circulatory hypoxia), and in conditions accompanied by an increase in oxygen demand (fever, pain, convulsions) [84, 90].

### Arterial-venous oxygen difference

This indicator was also described as an alternative trigger, but is less common in the literature. The arterio-venous oxygen difference is the difference between the oxygen content in arterial and venous blood:

5 $${\textbf{A}}-{\textbf{VO}}_{\textbf{2}}{\textbf{diff}}\hspace{0.17em}=\hspace{0.17em}{\textbf{CtaO}}_{\textbf{2}}-{\textbf{CtvO}}_{\textbf{2}}$$

In a prospective observational study, A. Fogagnolo et al. showed the benefits of using the arterio-venous difference as an additional blood transfusion trigger: in the group with high A-VO_2_ diff, blood transfusion was associated with a decrease in 90-day mortality [91]. However, another study devoted to this blood transfusion trigger has not been found.

### ECG signs of ischemia

One of the alternative blood transfusion triggers is ischemic signs on the electrocardiogram (ECG). The physiological rationale for this trigger is a decrease in oxygen delivery to the myocardium and consequently the development of myocardial ischemia [92]. Changes in the ECG are often functional and reversible and may be associated with tachycardia, but a number of authors indicate the possibility of using this feature as an additional physiological criterion for prescribing blood transfusion [93, 94]. Additionally, it is worth highlighting the low specificity of this trigger [73].

### Serum lactate

Another alternative trigger for blood transfusion might be the serum lactate level, which has been widely recognized as a reliable indicator of tissue hypoxia. A systematic review by A. Tran et al. showed the predictive value of lactate levels for transfusion and haemostatic interventions during traumatic hemorrhage [95]. On the other hand, there area large number of reasons for the rise in blood lactate levels, such as shock, sepsis, severe liver disease, which sharply reduce its specificity as a blood transfusion trigger [96, 97]. There are also no large randomized trials evaluating lactate as an indicator for blood transfusion.

It is important to note that changes in blood lactate levels and ECG during anemia often manifest late, which may lead to a delay in anemia treatment. Moreover, it has been shown that blood transfusion has no impact on reducing lactate levels, which may also limit its use as a trigger for blood transfusion [98].

### Near Infrared Spectroscopy

Near Infrared Spectroscopy is a non-invasive method for monitoring tissue oxygenation and can also be used as a physiological trigger for blood transfusion. In a systematic review published by Crispin and Forwood, 69 studies were included where Near Infrared Spectroscopy was employed to measure tissue oxygenation and guide decisions regarding blood transfusion. For certain patient groups (trauma patients), tissue oxygenation levels demonstrated low sensitivity and higher specificity for transfusion decisions. In other patient groups (cardiac surgery, neurosurgery, and neonatal cases), the use of Near Infrared Spectroscopy reduced the number of transfused units of red blood cells but did not impact outcomes [99]. The benefits of Near Infrared Spectroscopy in blood transfusion decision-making lie in its non-invasiveness; however, the available clinical data is insufficient to recommend it as a physiological trigger for blood transfusion.

### Exercise capacity

As mentioned above, many authors recommend considering not only the hemoglobin level but also clinical data, including a decrease in physical exercise tolerance, for blood transfusion decisions [32]. Cardiopulmonary exercises are typically used to assess exercise tolerance and are employed to stratify the risk of patients undergoing elective surgery. An increase in mortality and complications risk after surgery has been demonstrated in patients with reduced exercise capacity [100]. Some patients with anemia also exhibit decreased exercise capacity, which may be considered an additional trigger for blood transfusion. In a prospective clinical study, Wright et al. showed a significant improvement in exercise capacity after blood transfusion [101]. However, there is also insufficient data to recommend this method, and we suggest that it should be assessed in conjunction with other clinical data.

### Biomarkers of tissue hypoxia

A special interest in the study of anemia lies in markers of tissue hypoxia. Indicators such as methemoglobin and erythropoietin can serve as markers of anemia-induced tissue hypoxia. It is suggested that an increase in methemoglobin may be associated with an elevated risk of stroke, and assessing its level could be valuable in the management of anemia [102]. Erythropoietin has also shown an increase in response to tissue hypoxia and may be considered a marker of anemia-induced renal hypoxia [103]. However, further research on markers of anemia-induced tissue hypoxia and their role in the decision-making regarding blood transfusion is needed.

As seen from Table 4, current mainstream recommendations from professional medical societies for blood transfusion generally rely primarily on hemoglobin levels. At the same time, several alternative triggers for making decisions about blood transfusion have not gained widespread acknowledgment within these recommendations. However, many experts acknowledge the imperfections of hemoglobin levels as an isolated trigger for blood transfusion and the limitations in its application.

The recognition of the imperfections and limitations associated with relying solely on hemoglobin levels for blood transfusion decisions underscores the need for substantial clinical research into alternative transfusion triggers. Large clinical studies are crucial to comprehensively evaluate the effectiveness and safety of these alternative triggers, taking into account diverse patient populations and clinical scenarios.

A personalized strategy could consider not only hemoglobin levels but also other relevant clinical parameters, patient-specific factors, and physiological considerations. This personalized approach aims to optimize transfusion practices, ensuring a more tailored and effective intervention while minimizing unnecessary transfusions and associated risks.

In essence, the call for large clinical studies and the exploration of personalized approaches in the context of blood transfusion decisions stem from the recognition that a more nuanced understanding of patient needs and responses is essential to enhance the precision and appropriateness of transfusion practices.

**Table 4:** Red blood cell transfusion guidelines

Recommendations	Type of triggers recommended for use
Cross-Sectional Guidelines for Therapy with Blood Components and Plasma Derivatives [104]	Population: general patients Oxygen delivery indicators: - increasein oxygen extraction above 50%; - decrease in oxygen consumption by more than 10% - decrease in the level of mixed venous oxygen saturation less than 50% - decrease in the partial pressure of oxygen in mixed venous blood below 32 mm Hg - decrease in the level of central venous oxygen saturation less than 60% EKG signs: - ST segment changes; - new diagnosis of heart arrhythmia ; Clinical signs: - tachycardia; - hypotension; - dyspnea.
Russian Clinical guidelines for red blood cell transfusion [105]	Population: cardiologicalpatients: - increase in oxygen extraction above 50%; - decrease in the partial pressure of oxygen in the central venous blood below 32 mm Hg; - decrease in the level of mixed venous oxygen saturation less than 60% - an increase in the concentration of lactate in arterial blood to more than 2.5 mmol/l
Association of Anesthetists of Great Britain and Ireland: guidelines: the use of blood components and their alternatives 2016 [106]	Population: general patients Physiological triggers are not mentioned
Transfusion strategies in non-bleeding critically ill adults: a clinical practice guideline from the European Society of Intensive Care Medicine [33]	Population: non-bleeding critically ill patients Suggesting using hemoglobin or hematocrit transfusion triggers rather than physiologic transfusion triggers (conditional recommendation, very low certainty evidence).
Clinical Practice Guidelines From the AABB [32]	Population: general patients Physiological triggers are not mentioned, but attention is focused on the insufficiency of using the hemoglobin trigger as an indicator of oxygen delivery.
Practice Guidelines for Perioperative Blood Management: An Updated Report by the American Society of Anesthesiologists [107]	Population: patients in theperioperative period Physiological triggers are not mentioned

## Conclusion

Anemia is a fairly common clinical and hematological syndrome with a decrease in hemoglobin level as the main diagnostic criterion. Existing conservative drug treatments cannot always be used in patients in the perioperative period. The main treatment for anemia is blood transfusion, and the main trigger is the hemoglobin level. Sometimes additional triggers are used such as clinical signs of anemic syndrome. However, the clinical signs of anemia are not specific and cannot always be used in the clinical practice. Numerous studies have shown that both anemia and blood transfusion are independent factors that worsen outcomes.

The generally accepted consensus between these two problems is currently being solved by a restrictive transfusion strategy with reference points to a lower threshold hemoglobin level. However this strategy does not take into account some features of patients with comorbidities. As a result, studies on the threshold level of hemoglobin in patients with various acute diseases remain controversial. This fact has recently been emphasized by the results from the Myocardial Ischemia and Transfusion (MINT) trial, which evaluated whether a restrictive transfusion strategy (hemoglobin trigger, 7–8 g/dL) differed from a liberal transfusion strategy (hemoglobin trigger, < 10 g/dL) among patiens with acute myocardial infarction. Although the groups did not differ significantly regarding the primary composite outcome recurrent myocardial infarction or death at 30 days, the liberal treatment showed advantages in specific subgruoups [108]. This underlines again the importance of a multifactorial approach which addresses multiple patient specific risk factors and confirms the need for alternative blood transfusion triggers.

Despite the availability and interest in physiologic blood transfusion triggers, the level of evidence for their use is extremely low, which is primarily due to the lack of large randomized trials. At the same time, the need for personification of blood transfusion therapy is necessary. Vincent J.L., in one of his recent articles, gives a simple example: a hemoglobin level of 80 g/l is safe in a young healthy patient with an injury and the same hemoglobin level in an elderly patient with severe tachycardia and a history of myocardial infarction can play a critical role [36]. Current recommendations do not provide a clear solution to resolve this dilemma.

The application and study of alternative triggers for blood transfusion are crucial for several reasons. First, traditional triggers for transfusion, such as hemoglobin levels, may not fully capture an individual patient’s oxygen-carrying capacity and overall physiological status. By exploring alternative triggers, we can potentially identify more accurate and tailored indicators of when a patient truly requires a blood transfusion.

Second, the use of blood products comes with inherent risks, including transfusion reactions, infections, and immunological complications. By investigating alternative triggers, we can aim to reduce unnecessary transfusions, thus minimizing these potential adverse events and improving patient safety.

Moreover, studying alternative triggers can lead to more effective and efficient blood utilization, addressing the issue of blood shortages and ensuring the availability of blood products for patients who genuinely need them. This becomes especially critical in high-demand situations, such as during critical care or perioperative periods.

Furthermore, different patient populations may have distinct responses to anemia, making it essential to explore alternative triggers for blood transfusion in specific groups, such as critically ill patients or those undergoing surgical procedures. Tailoring transfusion practices based on individualized triggers may optimize patient outcomes and resource allocation.

In summary, the application and study of alternative triggers for blood transfusion are necessary to enhance patient care, minimize risks associated with transfusions, improve blood product utilization, and ultimately promote better outcomes in diverse clinical scenarios.

## Data Availability

Not applicable.
